# On the Equivalence of the Biological Effect Induced by Irradiation of Clusters of Heavy Atom Nanoparticles and Homogeneous Heavy Atom-Water Mixtures

**DOI:** 10.3390/cancers13092034

**Published:** 2021-04-23

**Authors:** Balder Villagomez-Bernabe, José Ramos-Méndez, Frederick J. Currell

**Affiliations:** 1 The Dalton Cumbria Facility and the School of Chemistry, The University of Manchester, Oxford Rd, Manchester M13 9PL, UK; frederick.currell@manchester.ac.uk; 2 Department of Radiation Oncology, University of California San Francisco, 1600 Divisadero Street, San Francisco, CA 94115, USA; jose.ramosmendez@ucsf.edu

**Keywords:** nanoparticle, cytoplasm, radiotherapy, nanomedicine

## Abstract

**Simple Summary:**

The use of nanoparticles in radiotherapy has been studied widely for over a decade due to their ability to reduce the survival fraction of tumor cells while reducing the doses deposited in healthy cells. Mathematical models were successfully implemented to reproduce experimental results in the literature at the keV range, but discrepancies were found at the MV energy range, the latter range being most used in radiotherapy. The main finding of this work is the demonstration of an equivalence of the physically mediated component of the cell damage between a cluster of nanoparticles and a gold–water mixture in the MV energy range, which reduces the complexity of modeling the interactions of radiation with clusters of nanoparticles seen in real case scenarios.

**Abstract:**

A multiscale local effect model (LEM)-based framework was implemented to study the cell damage caused by the irradiation of clusters of gold nanoparticles (GNPs) under clinically relevant conditions. The results were compared with those obtained by a homogeneous mixture of water and gold (MixNP) irradiated under similar conditions. To that end, Monte Carlo simulations were performed for the irradiation of GNP clusters of different sizes and MixNPs with a 6 MV Linac spectrum to calculate the dose enhancement factor in water. The capabilities of our framework for the prediction of cell damage trends are examined and discussed. We found that the difference of the main parameter driving the cell damage between a cluster of GNPs and the MixNP was less than 1.6% for all cluster sizes. Our results demonstrate for the first time a simple route to intuit the radiobiological effects of clusters of nanoparticles through the consideration of an equivalent homogenous gold/water mixture. Furthermore, the negligible difference on cell damage between a cluster of GNPs and MixNP simplifies the modelling for the complex geometries of nanoparticle aggregations and saves computational resources.

## 1. Introduction

Nanoparticles of high atomic number have been widely used in medicine as contrast agents and nano-carriers due to their biocompatibility and small size [1,2]. Furthermore, since Hainfeld’s studies in 2004 on the use of nanoparticles as radiosensitizers [3], considerable effort has been made to understand the mechanisms of physical interactions between ionizing radiation and nanoparticles. The role of such nanoparticles in biomedical imaging, cancer therapy, and biophysics has been reviewed elsewhere [4,5]. Theoretical models [6,7] have predicted that the maximum physical enhancement should be seen at keV energies, where the highest values for the ratio of the mass energy absorption coefficient between gold and water were calculated. At that energy range, the photoelectric effect is the predominant physical process, which leads to the generation of a cascade of low energy Auger electrons during the deexcitation process after each interaction; those Auger electrons are mainly responsible for depositing a high quantity of energy around the nanoparticle, seen as spikes of localized dose in the cell near or hosting the nanoparticle.

Nevertheless, as Butterworth et al. [8] have summarized, previous theoretical models failed to predict the radiosensitization enhancement observed in experiments in vitro for MV irradiation. Those models assumed (implicitly) a uniform distribution of the nanoparticles inside the cells, neglecting mutual interactions between them. However, it has been shown that nanoparticles tend to aggregate inside the tumor [9,10,11], leading to heterogeneous distributions even inside the cells, suggesting the necessity for further studies on the change in radiobiological response due to clustering of nanoparticles. We therefore applied the local effect model to perform a multiscale approach under relevant clinical conditions in order to investigate the radiosensitization enhancement in cells due to clusters of gold nanoparticles (GNPs) of different sizes and positions relative to the radiation-sensitive structures inside the cell.

The effects of nanoparticles on DNA are currently a matter for discussion in the scientific literature, see e.g., [12,13]. Furthermore, it is not clear that the only critical subcellular target is nuclear DNA. Possible critical targets located in the cytoplasm include mitochondria and lysosomes [14,15].

**Figure 1:**
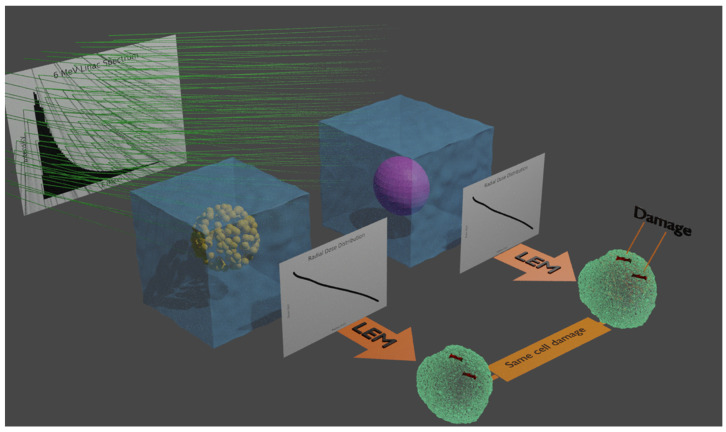
The histogram at the back shows the 6 MV Linac spectrum used to irradiate both a cluster of gold nanoparticles (GNPs) and the MixNP (homogeneous mix of gold and water) in order to calculate radial dose distributions (RDDs) in water (black curves). The cluster of GNPs is represented by many small gold spheres in the left-most water cube, whilst the gold-water mixture comprising the MixNP is represented by the magenta sphere in the right-most water cube. The RDDs were inserted into the local effect model (LEM) framework, represented as orange arrows, for the calculation of the biological effect.

Due to the complexity of GNP distribution inside the tumor cell, previous works in the literature [16,17] have approximated the clusters with a homogeneous mixture of water and gold. A review of recent progress in gold nanoparticle dosimetry can be found in [18]. To the best of our knowledge, no benchmarking against the presence of clusters of GNPs has yet been carried out to compare the effects at the biological stage. Thus, in this work we carried out a set of Monte Carlo simulations to test the hypothesis of the equivalence of the biological effect generated by dose enhancement at the physical stage between clusters of nanoparticles and homogenous mixtures. The concepts underlying this comparison are shown in Figure 1.

## 2. Materials and Methods

The multiscale approach performed in this work involved the prediction of the biological effect from the interactions between ionizing radiation and GNPs/MixNP. Although the calculations presented are concerned with GNPs, the modelling of physical processes will be similar for any nanoparticle containing a significant fraction of heavy atoms. Hence, the results are applicable to this wider class of nanoparticles.

### 2.1. Physical Stage

We performed a set of Monte Carlo simulations consisting of condensed-history Monte Carlo with Geant4, suitable for macroscale; and track-structure Monte Carlo with Geant4-DNA, suitable for micro and nanoscale. The different physics lists and relevant parameters for the electromagnetic processes used in each simulation are shown in Table 1. Deexcitation processes including Auger, Auger cascade, and fluorescence, were activated for all simulations.

The simulation at the macroscale involved the transport of X-rays through a water phantom of 20 × 20 × 40 cm^3^. To that end, the initial energy spectrum was taken from a 6 MV TrueBeam Linac set in Flattening-Filter mode to produce a 10 × 10 cm^2^ field at 90 cm source-surface distance (SSD), obtained elsewhere [19]. The X-rays traveled in the water phantom and a phase space file (PHSP1) was generated in a circular plane of 0.25 cm radius, placed at the depth of the maximum dose. The phase space file stored the information of all particles, charged and neutral, crossing that plane. For the simulation at the nanoscale, the spatial coordinates of the phase space were scaled down to the nanoscale as explained in [20]. Later, the PHSP1 was used as a particle source to irradiate the cluster of nanoparticles and the mixture of water and gold. In order to reduce statistical uncertainty for the radial dose distribution (RDDs) below 2% for all radial bins, the phase space file was recycled 30, 50, and 200 times for the 500, 250, and 70 nm clusters, respectively. 

The three cluster sizes considered in this work consisted of 333 GNPs of 7, 25, and 50 nm radius. The GNPs were distributed in a randomly uniform distribution without overlapping, ensuring that all of them were all bounded inside a big water nanoparticle (WNP) of 70, 250, and 500 nm radius, respectively. After irradiating the cluster using PHSP1, a new phase space (PHP2) was recorded at the outer surface of the WNP with the information of only those electrons that were created inside the cluster and able to escape from it. The simulations for the full cluster were repeated ten times using different random seeds.

Three MixNPs were modelled as spheres of radius 70, 250, and 500 nm made of a homogeneous mixture of gold and water with density of 7.1 g/cm^3^. The density was calculated, taking into account the mass of water and gold inside the cluster over the entire volume. We performed the simulations using the PHSP1 as a source to irradiate the MixNPs, generating a new PHSP2, similarly to the process used for the full cluster. Ten simulations were run using different random seeds.

Lastly, two independent track–structure simulations were performed using the Geant4-DNA physics list [21,22,23] to calculate the dose enhancement in water by GNPs and MixNP activation using their corresponding PHSP2s, as calculated previously. Each PHSP2 was positioned at the center of a water sphere of 1 mm radius. Multiple concentric spherical scorers were placed to record RDD in the sphere. To speed up the simulations, the thickness of the spherical scorers was set as shown in Table 2. Ten simulation jobs were launched for both the MixNP and the cluster of GNPs in order to obtain the mean value of the RDDs with their associated standard error.

### 2.2. Biological Stage

We modelled a biological cell as two concentric spheres of 15 and 7 µm radius, representing the cell and nucleus, respectively. The placement of the cluster varied from 7 to 40 µm from the nucleus surface to study cell damage as a function of the relative distance between the cluster/MixNP and the volumes of interest. The lower limit was chosen to avoid the placing of the cluster inside the nucleus, while the upper limit was set to study how cluster distance influences damage to nearby cells.

### 2.3. Software and Hardware Specifications

The software used to perform the Monte Carlo simulations of the transport of ionizing radiation through matter was TOPAS (Tool for Particle Transport Simulation) [24] version 3.2, which is built on top of the Geant4 toolkit [25] version 10.5. TOPAS wraps and extends Geant4 with a simplified syntax. 

The Monte Carlo simulations were carried out using the HPC Pool facilities at the University of Manchester with the following specifications: 128 × 32 compute nodes, each node involving 2 × 16-core Intel Skylake Gold 6130 CPUs at 2.10 GHz and 192 GB RAM.

## 3. Results

We studied the difference in cells damage derived from dose enhancement by a cluster of GNPs and a gold/water mixed nanoparticle (MixNP) activated with X-ray irradiation. To compare the differences in the physical and biological stages between them, the percentage of the difference *(%Diff)* of all the parameters of interest were calculated using Equation (1).

*%Diff* = (*X_mixNP_* − *X_cluster_*) ∗ 100/*X_cluster_*, (1)

where *X_mixNP_* and *X_cluster_* represent any pertinent observable associated with the MixNP and a cluster of GNPs, respectively. Thus, a negative percentage difference suggests a higher value of the observable for the cluster than that for the MixNP. 

### 3.1. Physical Stage: The Monte Carlo Simulations

**Figure 2:**
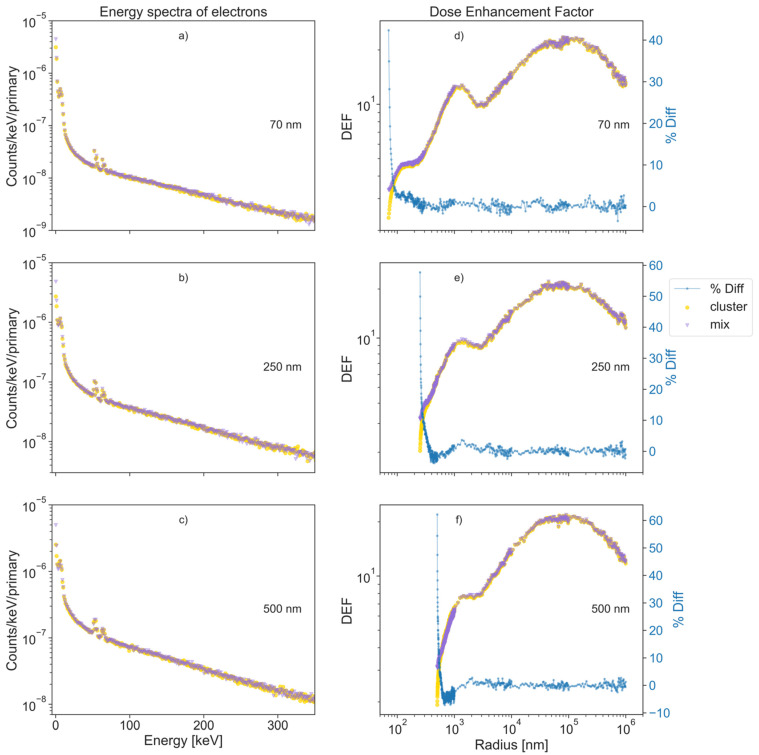
Three different cluster sizes of 70, 250, and 500 nm, (**a**–**f**) each made of 333 GNPs of 7, 25, and 50 nm radii, respectively (top to bottom), were irradiated with a 6 MV energy spectrum from a TrueBeam Varian Linac. Left columns (**a**–**c**) show the energy spectra of electrons created inside the cluster and a MixNP that escaped from it (yellow and purple, respectively), for all cluster sizes. Here, the highest difference between both spectra is at the first energy bin, which corresponds to energies below 0.1 keV. In the right column, (**d**–**f**) show the DEF of the MixNP and the full cluster, and also the *%Diff* (in blue) for a better visualization of the differences on the DEF for all sizes. Here, we observed that a higher number of low-energy electrons from the MixNP leads to an enhancement of the DEF at small radii, compared to the DEF of the cluster. The high values of the *%Diff* at small radii are associated to the models used to simulate full clusters, where the gold concentration decreases at the surface of the cluster. It is shown in the Supplementary Material that the values of the *%Diff* were reduced dramatically when a more complex MixNP was modelled as two concentric spheres made of water and a mix of gold plus water for the outer and the inner sphere, respectively.

The results of the physical stage obtained through Monte Carlo simulations involving the interaction of a cluster of GNPs with X-ray irradiation are shown in Figure 2. The left column shows the energy spectra of the secondary electrons that were created inside the cluster and were able to exit from it, for the three different cluster sizes (70, 250, and 500 nm). The right column illustrates a comparison to the dose enhancement factors (DEFs). For any set of conditions, the DEF is defined in Equation (2) as the ratio of the dose deposited in the water phantom measured in a given radial bin over the dose in the same radial bin when the cluster/MixNP is replaced with a water nanoparticle of the same size, i.e.,

*DEF*(*r*) = *Dose_GNP_*(*r*)/*Dose_WNP_*(*r*), (2)

where, *Dose_GNP_*(*r*) and *Dose_WNP_*(*r*) are the dose deposited in the radial bin *r* by a cluster/MixNP and a WNP, respectively. 

The left column in Figure 2 shows that the energy spectrum of the secondary electrons leaving the cluster was very similar to that escaping from the MixNP, except at lower energies. The maximum difference was found in the first bin, centered at 0.1 keV, where MixNP produced 42, 77, and 97% more electrons than a full cluster for the radii of 70, 250, and 500 nm respectively. For better visualization, the energy spectrum was plotted up to 350 keV, as only a low percentage of electrons have energies greater than that value (3.7, 6.7 and 8.6% for the cluster sizes of 70, 250, and 500 nm radii, respectively). 

The right column in Figure 2 illustrates the influence the percentage difference of the energy spectrum of electrons at low energy, as mentioned above, has on the dose enhancement factor (DEF), in water. In the figure, it is observed that the higher values of the *%Diff* for the DEF occurred at shorter radial distances. The maximum difference occurred at the first radial bin, where it took values of 42, 58, and 62%, and decreased sharply down to 5% at 15, 55, and 50 nm for the 70, 250, and 500 nm cluster sizes, respectively. For better visualization of such difference, we plotted the *%Diff* obtained from Equation (1). The high values of the *%Diff* at small radius can be associated with the geometrical model used to simulate the full cluster, where all the gold nanoparticle positions were fixed to be entirely inside the boundary of the cluster. This condition led to a reduction in the average amount of gold material near the surface of the cluster, in contrast with the constant gold density of the MixNP. This effect is illustrated in Figures S1–S3, in the Supplementary Materials, where MixNP was modelled as two concentric spheres made of water and a mix of gold plus water for the outer and inner spheres, respectively, using the parameters in Table S1, leading to a considerable decline of the *%Diff* near the cluster surface. However, when the biological effects are considered, there is no benefit to using this more complicated structure for the MixNP, so a simple homogenous spherical geometry was used for the majority of this study. The “waviness” of the DEF was directly correlated to the energy of the secondary electrons produced by the photoelectric effect inside the nanoparticle [26], which not only generated photoelectrons but also initiated an Auger cascade of low-energy electrons during the atomic deexcitation. 

### 3.2. Biological Stage: The Local Effect Model

We applied the local effect model described in [27] to predict the average number of lethal lesions (*N_to_*_t_) in the cell, as shown below:

(3) $$\langle N_{total}\rangle =\alpha +\beta D^{2}+\left(\alpha DI_{1}+\beta DI_{2}+2\beta D^{2}I_{1}\right)/V_{sens},$$

where α and β are experimental parameters that depend on the cell line, while *D* represents the dose deposited in point *r* (in the absence of GNPs), and *V_sens_* is the sensitive volume over which the lethal lesions are calculated. Here, *I*_1_ and *I*_2_ are the main parameters for the prediction of the *N_to_*_tal_ due to nanoparticle activation with X-rays. By considering the cell as two concentric spheres (cytoplasm and nucleus), where a spherical cluster of nanoparticles is placed inside the cytoplasm, we can exploit the symmetry of this system and approximate *I*_1_ and *I*_2_, as in [28]:

(4) $$I_{1}=\int^{\;}D\left(r\right)dV\;\approx \int^{\;}\frac{\Omega \left(r\right)}{4\pi }S_{1}\left(r\right)dr,$$

(5) $$I_{2}=\int^{\;}D^{2}\left(r\right)dV\approx \;\int^{\;}\frac{\Omega \left(r\right)}{4\pi }S_{2}\left(r\right)dr,$$

where the function Ω(r) represents the solid angle between the center of the cluster/MixNP and the volume of interest at the radial distance *r*. Here, we neglected the dependency on the angles because the cluster has no specific orientation within the volume of interest or radiation field. Here *S*_1_ and *S*_2_ are defined as:

(6) $$S_{1}\left(r\right)=\int_{S}D\left(r\right)dA,$$

(7) $$S_{2}\left(r\right)=\int_{S}D^{2}\left(r\right)dA.$$

Equations (6) and (7) were solved numerically to calculate *S*_1_ and *S*_2_, where the RDDs scored from the Monte Carlo simulations were used as input parameters. Ω(r) was calculated analytically for the two regions of consideration (nucleus and cytoplasm) by exploiting the spherical symmetry of the cell, as explained in [28]. Parameters *I*_1_ and *I*_2_ were calculated for both regions of interest by substituting *S*_1_, *S*_2_, and Ω(r) in Equations (4) and (5) and solving them numerically. We repeated the same procedure for the MixNP in order to obtain the percentage difference (*%Diff*) between them, using Equation (1) to investigate the difference in cell damage caused by the cluster of GNPs with respect to the MixNP. 

**Figure 3:**
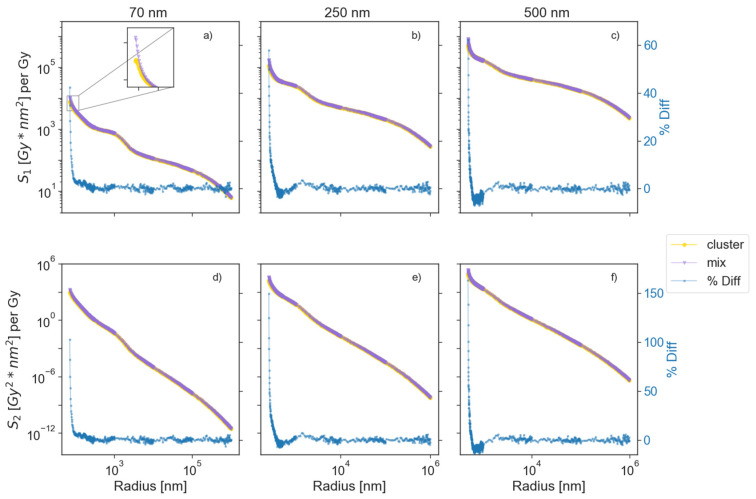
Upper row (**a**–**c**) shows the behaviors of *S*_1_ for a cluster of GNPs and a MixNP for all sizes, along with the *%Diff* between them (blue). *S*_1_ represents the dose in a spherical shell and thickness of *dr* integrated over the surface of a circle of radius *r*. Due to the slow decrease of *S*_1_ along the radius, we scored the dose far away from the nanoparticle. Lower row (**d**–**f**) shows the change in *S*_2_ along the radial distances. *S*_2_ is defined similarly to *S*_1_, but using the dose squared in the surface integral. Due to the closeness of the values of *S*_1_ for the full cluster and a MixNP, a zoomed-in view was added in (**a**).

Figure 3 shows the behavior of the parameters *S*_1_ and *S*_2_ defined in Equations (6) and (7). A steady decrease of *S*_1_ (associated with the *r*^2^ dependence of the area), led to a necessity to score the dose deposited in the cell at far distances from the cluster. The highest values of the *%Diff* for *S*_1_ in the first radial bin were 42, 57, and 62% (smallest to largest clusters). However, this difference quickly fell to 5% for the radial distances of 15, 60, and 50 nm beyond the cluster surfaces for the 70, 250, and 500 nm sizes, respectively. The highest values of the *%Diff* for *S*_2_ were 102, 149, and 163% in the first radial bin. These differences fell to 5% at 26, 80, and 73 nm beyond the cluster surfaces for the of 70, 250, and 500 nm sizes, respectively. 

In this work, the cell was approximated as two concentric spherical regions (nucleus and cytoplasm). Our model allowed for a study of the change in the average number of lethal lesions when the cluster/MixNP was placed at different positions inside the cell. Furthermore, our model also makes it feasible to consider both regions as our sensitive volume. Nevertheless, previous studies where GNPs were injected into several cell lines [29,30], associate a low probability to nanoparticle entry into the nucleus, therefore, this study only comprised of placements of the nanoparticle clusters outside the nucleus. More recent imaging studies [31] have shown that monodispersed nanoparticles can in fact penetrate the nucleus. In this case, however, it is clear that the approximation used here will break down since it considers the cluster of nanoparticles to be biologically inert. For that case, a more complex formalism has been already provided [28], which can be used to predict dose enhancements, as has already been illustrated [31]. In addition, clusters/MixNPs were also considered to be far outside the cell in order to study the influence of nanoparticles placed inside the surrounding cells on the lethal lesions created in the *V_sens_*. 

**Figure 4:**
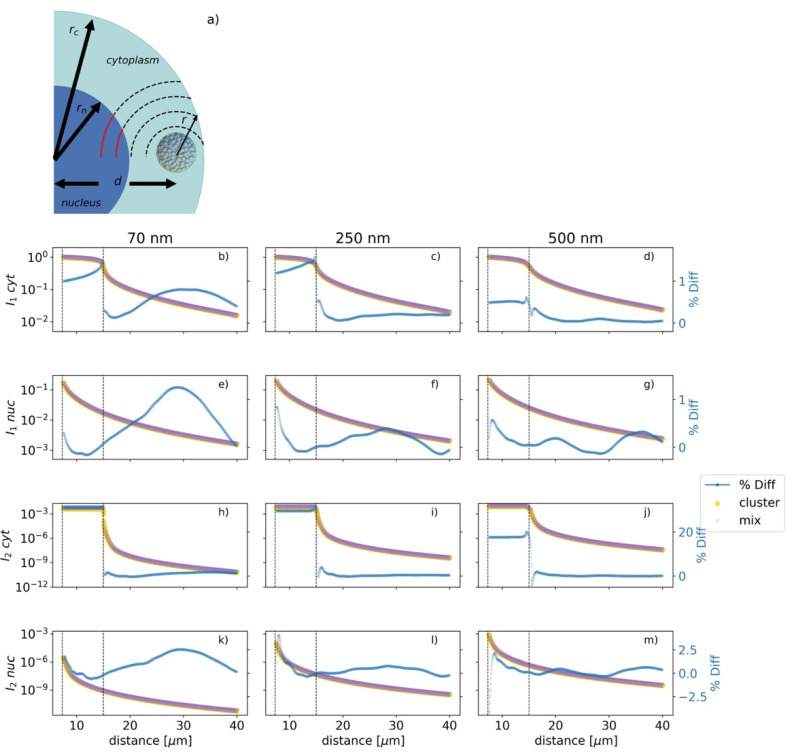
(**a**) The cell used in this work was modelled as an outer and an inner sphere representing the cytoplasm and nucleus, with radii of *r_c_* = 15 and *r_n_ =* 7 µm, respectively. The cluster/MixNP was placed at different positions *d* from the center of the cell for the evaluation of *I*_1_ and *I*_2_ for all cluster sizes. (**b**–**g**) show *I*_1_ for a MixNP and a full cluster along with the *%Diff* between them when considering the cytoplasm and the nucleus as *V_sen_*_s_, respectively. (**h**–**m**) describe *I*_2_ for a MixNP and a full cluster as well as the *%Diff* between them when considering the cytoplasm and the nucleus as *V_sens_,* respectively. All the curves in the same column are normalized with respect to the *I*_1_ *cyt* for the cluster of GNPs. The two black dotted lines correspond to the distances of 7.5 and 15 µm.

When *V_sens_* was assigned to the cytoplasm, the parameters of interest in the biological stage were *I*_1_ *cyt* and *I*_2_ *cyt*, but those changed to *I*_1_ *nuc* and *I*_2_ *nuc* when the nucleus was considered as *V_sens_*. Figure 4 shows the changes of *I*_1_ and *I*_2_ when the cluster/MixNP was placed at different locations inside the cell. Figure 4b–d,h–j consider the cytoplasm as *V_sens_*, while Figure 4e–g,k–m consider the nucleus as the *V_sens_*. Figure 4 also shows the *%Diff* between the parameters of interest for all scenarios. 

As shown in Figure 4b–d, we observed that the *%Diff* for the *I*_1_ *cyt* rose between the distances of 7 and 15 µm (the nucleus and cytoplasm radii, respectively), and reached a peak at 15 µm, with values of 1.52, 1.53, and 0.62 for the cluster sizes of 70, 250, and 500 nm, respectively. This peak corresponded to the cluster spanning the cell membrane and hence does not represent a physically realistic scenario. Figure 4h–j shows that the *%Diff* for the *I*_2_ *cyt* remained steady inside the cell and that its peak values at the cell edge were 32.7, 32.8, and 20.2 for the cluster sizes of 70, 250, and 500 nm, respectively, dropping sharply after that. *I*_1_ *cyt* had a steady decline inside the cell volume for the MixNP and the full cluster and declined at a faster rate beyond the cell boundary. *I*_2_ *cyt* remained steady inside the cell and plummeted at 15 µm, decreasing for further distances in both the MixNP and the full cluster scenarios.

Figure 4e–g,k–m illustrate the parameters of interest when the nucleus was considered as the sensitive volume. The position of the maximum values for the *%Diff* for the *I*_1_ *nuc* varied between cluster sizes, being localized at 28.85, 7.55, and 8.10 µm for the 70, 250, and 500 nm cluster sizes, respectively; while the maximum values for the *%Diff* for the *I*_2_ *nuc* were 29.4, 0.8, and 8.55 µm for the 70, 250, and 500 nm sizes, respectively. *I*_1_ *nuc* and *I*_2_ *nuc* decreased steadily for all distances, having their maximum at the nucleus surface.

## 4. Discussion

In this work, we investigated the differences in the cell damage generated by the activation of a cluster of gold nanoparticles irradiated by a 6 MV TrueBeam Varian Linac compared to a homogeneous mixture of gold and water irradiated under the same conditions. The data from Figure 4 illustrate that for both scenarios, the cluster of GNPs and the MixNP, the main parameter driving the biological stage for all cluster sizes was *I*_1_, which is several orders of magnitude higher than *I*_2_. This was true for both of the sensitive volumes considered in this work: the cytoplasm and the nucleus. Furthermore, *I*_1_ *cyt* was one order of magnitude higher than *I*_1_ *nuc*, implying that the majority of the energy deposition occurred outside the nucleus. Thus, by neglecting *I*_2_ from Equation (3) and rearranging the terms, we can define the radio sensitizer enhancement factor (*SEF*) as:

(8) $$SEF=I_{1}\frac{\alpha D+2\beta D^{2}}{V_{sens}\left(\alpha D+\beta D^{2}\right)}$$

where α and β are constant parameters depending on the cell line, while *V_sen_*_s_ depends on the sensitive volume of the cell in which we are counting the number of lethal lesions, leaving *I*_1_ as the main parameter driving the change to the surviving fraction generated by the nanoparticle.

The high value of the *%Diff* at the physical stage shown in Figure 2 translates into the biological stage as high values *%Diff* values for *I*_2_ *nuc* and *I*_2_ *cyt*, as illustrated in Figure 4. However, *I*_1_ *cyt* drives the biological stage, as mentioned above. The *%Diff* fell to ~1.5% for the 70 and 250 nm clusters and fell to less than 1% for the 500 nm cluster. Therefore, the number of lethal lesions in the cell created by the activation of a full cluster was satisfactorily approximated by the MixNP. This novel result implies two things: firstly, it provides a ready reckoning of how uptake in a microscope image (without resolving individual nanoparticles) can be related to enhancement, and secondly, it facilitates the reduction of the many-body problem to one body when considering the interior of the cluster as not biologically active, considerably reducing the complexity of the problem and saving computational resources. This study estimates a 50% reduction of computational time for the Monte Carlo simulations at nanoscale when a considering a MixNP instead of a full cluster of GNPs for calculations of PHSP2. 

Although our simulations were confined to spherically symmetric clusters, this was simply for computational convenience, as it provided simple closed forms for the relationships expressed in Equations (4)–(7). Our main conclusion is that using the homogenous MixNP results in the same predicted biological effects as the cluster of individual NPs, provided the effect is being considered outside of the cluster. It is immediately obvious that this result extends to any shape of cluster since it can be well represented by an ensemble of spherical clusters, i.e., there is a super-position principle one can apply to the evaluation of *I*_1_ and *I*_2_*,* approximating any shape of cluster by means of many spherical ones.

The increase of the *%Diff* between a cluster and a MixNP near the nanoparticle surface in the physical stage, as shown in Figure 2, was associated with the simplified modelling for the MixNP as a perfectly homogeneous mix, causing an excess of electrons at the surface. By contrast, with a real cluster the average density decreases slightly at the surface due to the amount of interstitial water between the nanoparticles. The high values of the DEF created by the MixNP agree with previous work by Zhan and Koger [32,33], who obtained ~30% for both studies using mono-energetic gamma beams. Nevertheless, by investigating this phenomenon in more detail, we observed that more complex models for the MixNP could decrease the *%Diff* considerably at the physical stage, as is shown in the Supplementary Materials. This quantity decreased by approximately one order of magnitude by considering a water shell surrounding the MixNP. However, the use of more complex models was not necessary for this work because the *%Diff* for the biological *I*_1_ became negligible when the LEM was applied and integrated over the sensitive volume. 

Due to the current lack of knowledge on the radiolysis when GNPs are present during irradiation, the chemistry stage was out of scope in this work. However, we cannot discard the possibility of a higher value of the *%Diff* for *I*_1_ and *I*_2_ in the biological stage when the production of radicals is taken into account. 

Previous experiments support the evidence that the nanoparticles tend to cluster inside the cell [9,10,11], suggesting this study represents a more realistic scenario than those interpreting GNPs with a uniform random distribution inside the cell. Furthermore, the approach used to model the aggregation of nanoparticles using a spherical geometry is supported by previous work in the literature [34] where the sizes of the clusters were measured by their diameters using TEM scan. 

The maximum value for the DEF observed in this work at the physical stage (~20) when the GNPs of all cluster sizes were activated with 6 MV Linac irradiation is in agreement with previous studies [35,36], where the maximum values of the DEF were found to be between 8 and 17. The dependency observed on the relationship between the position of the cluster inside the cell and the enhancement of DNA double-strand break (DBS) formation inside the nucleus during irradiation was also observed in previous work based on computational simulations [28,37]. In these studies, the cells were irradiated under similar conditions to work. Furthermore, the results of [38,39,40], where various cell lines were investigated using different techniques such as radiation-induced foci of γ-H2AX staining and immunohistochemistry, confirm the trend for *I*_1_ *nuc*, as shown in Figure 4, to damage cell nuclei at mega-voltage energies.

Clearly, for the model presented here to be valid, the size of each nanoparticle needs to be significantly smaller than the overall size of the cluster or, equivalently, there needs to be many nanoparticles in the cluster. Provided this condition is met, there is an interesting and perhaps slightly counterintuitive conclusion—the biological effect is only very weakly dependent on the nanoparticle size (see Figure 4). It should, however, be noted that we factored the dominant nanoparticle size dependence out of our simulations, since we constructed clusters containing a constant number of nanoparticles and a constant total fractional density of gold, i.e., bigger nanoparticles were used to constitute bigger clusters.

Although the model presented is a macroscopic one, it pertains at the single-cell level, as illustrated in Figure 4. Hence, it is applicable at either the cellular level or at the patient/organ level when applied to radiotherapy. However, in the former case, the validity of the model rests on the assumption that the biologically active region is entirely outside the cluster. This assumption is not always valid, e.g., see [31].

The similarities between the *I*_1_ curves for both the MixNP and the full cluster for cases where the critical target was either in the nucleus (*I*_1_ *nuc* of Figure 4) or the cytoplasm (*I*_1_ *cyt* of Figure 4) show that that there was an equivalence of the biological effect induced by irradiation of clusters of heavy-atom nanoparticles and homogeneous heavy-atom–water mixtures for either type of target. Furthermore, one could take a linear combination of *I*_1_ *nuc* and *I*_1_ *cyt* corresponding to the different weighting factors for the nuclear and cytoplasmic targets and still have nearly equivalent results for the MixNP and full cluster cases. Hence, the conclusion regarding the equivalence of the biological effect in these two scenarios is robust, even though there is ongoing controversy regarding the nature of the critical targets, as was highlighted in the introduction [12,13,14,15].

## 5. Conclusions

We implemented a multiscale approach by applying the LEM to fully simulated clusters of GNPs using the Monte Carlo technique and irradiated them in a clinically relevant scenario. The resultant biological effect in cells is well represented through a single parameter, *I*_1_, which describes the increase in the number of lethal lesions for the two sensitive volumes studied in this work. The predicted cell damage generated by such clusters is essentially the same as for a homogeneous mixture of gold and water. This equivalence is useful both to intuiting the effect of measured nanoparticle distributions and also to performing a rapid computational evaluation of these effects.

## Figures and Tables

**Table 1:** Parameter values for each simulation performed at the physical stage.

Parameters	MC Condensed History		MC Track-Structure
Physics list	Livermore	Livermore	Geant4-DNA
Energy cut (eV)	100	10	10
Range cut (nm)	1000	1	1
Max step (nm)	1000	1	1
Used for calculating	PHSP1	PHSP2	RDD

**Table 2:** Bin sizes used for the calculation of RDDs in water for each cluster size.

Bin Size [nm]	Range [nm] for Each Cluster Size		
	70 nm	250 nm	500 nm
1	70–300	250–500	500–1000
10	300–1 × 10^3^	500–1 × 10^3^	–
100	1 × 10^3^–1 × 10^4^	1 × 10^3^–1 × 10^4^	1 × 10^3^–1 × 10^4^
1 × 10^3^	1 × 10^4^–1 × 10^5^	1 × 10^4^–1 × 10^5^	1 × 10^4^–1 × 10^5^
1 × 10^4^	1 × 10^5^–1 × 10^6^	1 × 10^5^–1 × 10^6^	1 × 10^5^–1 × 10^6^

## Data Availability

Computer codes used to perform these calculations will be made publicly available through the University of Manchester’s institutional repository upon its acceptance for publication.

## Supplementary Materials

More information on the modelling of the complex MiXNP mentioned in Results section is available online at https://www.mdpi.com/article/10.3390/cancers13092034/s1, Figure S1: Visualization of the heterogenous MixNP, Figure S2: DEF and *%Diff* (blue) between the cluster and the heterogenous MixNP for all sizes, Figure S3: Box plot comparing the %Diff between the cluster and the heterogeneous/homogeneous MixNP for all sizes, Table S1: Set of optimized parameters used to model the heterogeneous MixNP for all sizes.
