# Supplementary material for: On the Equivalence of the Biological Effect Induced by Irradiation of Clusters of Heavy Atom Nanoparticles and Homogeneous Heavy Atom-Water Mixtures
Source: Cancers (Basel). 2021 Apr 23;13(9):2034. doi: 10.3390/cancers13092034 (PMC8122863; doi:10.3390/cancers13092034)
Supplement: Supplementary file 1 [file cancers-13-02034-s001.zip › cancers-1182992-supplementary.pdf]

Supplementary Material

Here we explore the *%Diff* at the physical stage between a cluster of GNPs and an heterogenous MixNP, modelled as a two concentric spheres as shown in Fig. S1, where the gold/water mixture is surrounded by water cell.

The seeting of the properties of the inner sphere for the heterogeneous MixNP were set *a posteriory*, by sampling it's density and radius, and choosing the parameters that minimizes the *%Diff* of the Radial Dose Distributions (RDDs) with respect to the GNPs cluster. The best set of parameters are shown in Table S1. Using those values, the DEF and %Diff for the all sizes of the heterogenous MixNP are plotted in Fig. S2.

To show the drastic improvement on the %Diff by the hereogenous MixNP, Fig. S3 shows a comparison of the %Diff between a full GNPs cluster versus the homogeneous and heterogeneous MixNP.

Table S1. Set of optimized parameters used to model the heterogeneous MixNP for all sizes.

MixNP size (nm)	Density (g/cm3)	Core radius (nm)
70	8.767	64
250	7.895	240
500	7.9	480

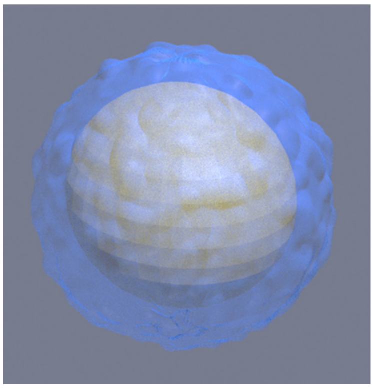

Figure S1. Visualization of the heterogenous MixNP, showing a water cell surrounding an homogeneous mixture of gold and water.

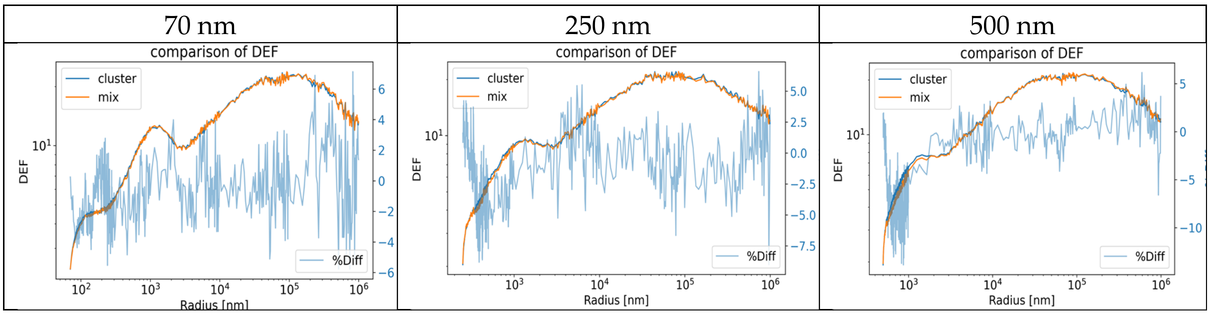

Figure S2. DEF and %Diff (blue) between the cluster and the heterogenous MixNP for all sizes.

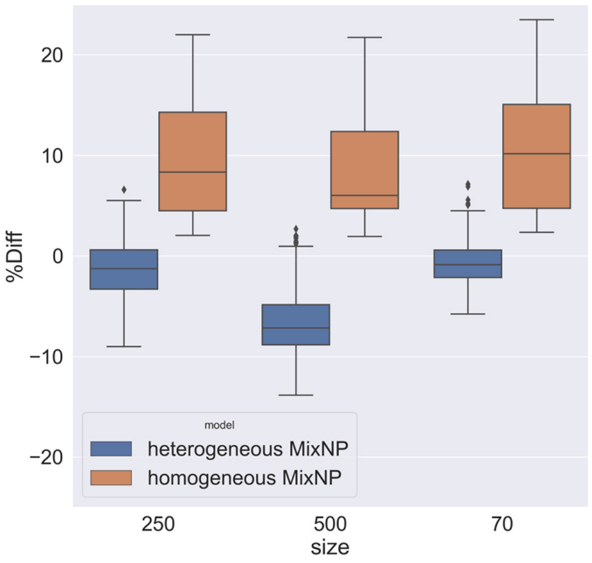

Figure S3. Boxplot comparing the %Diff between the cluster and the heterogenous/homogeneous MixNP for all sizes.
